# *Staphylococcus epidermidis*-associated brain abscess following endovascular treatment of a ruptured intracranial aneurysm: potential immune-inflammatory mechanisms—a case report and literature review

**DOI:** 10.3389/fimmu.2026.1974345

**Published:** 2026-10-27

**Authors:** Xufeng Sun, Qile Sun, Shenao Li, Jiahao Zhang, Xiaolin Xu, Yueyang Peng, Zhifeng Wen

**Affiliations:** 1 Department of Neurosurgery, The First Hospital of China Medical University, Shenyang, China; 2 School of Public Health, China Medical University, Shenyang, China; 3 Department of Neurosurgery, Ledong Li County People’s Hospital, Ledong, China

**Keywords:** blood-brain barrier, brain abscess, endovascular treatment, *Staphylococcus epidermidis*, subarachnoid hemorrhage

## Abstract

**Background:**

Endovascular treatment(EVT) is a key therapeutic approach for ruptured intracranial Aneurysms; however, postoperative brain abscesses are extremely rare, and their pathogenesis and optimal treatment strategies remain unclear. This article reports a case of a *Staphylococcus epidermidis*-associated brain abscess that developed following stent-assisted coil embolization for subarachnoid hemorrhage caused by a ruptured anterior communicating artery aneurysm and discusses the underlying immune and inflammatory mechanisms as well as treatment strategies.

**Case presentation:**

A 49-year-old man underwent stent-assisted coil embolization for subarachnoid Hemorrhage(SAH) from a ruptured anterior communicating artery aneurysm. Recovered well from operation and discharged. He, however, presented on the 34th postoperative day with a high-grade fever, headache, nausea, and vomiting. Brain imaging showed a brain abscess in the left frontal lobe, approximately 42 mm × 27 mm. However, there were no clear signs of severe neurological deterioration or a critical phase of cerebral herniation. Laboratory tests revealed high white blood cell and neutrophil counts, low lymphocyte counts, and variable reductions in CD3+, CD4+, and CD8+ T cells, CD19+ and CD20+ B cells, and Natural Killer cells(NK cells). Meanwhile, IL-1β, IL-6, TNF- α, and IL-10 levels increased, indicating deep immunological and inflammatory changes during infection. Inflammatory and immune markers improved slightly after antimicrobial therapy, but clinical symptoms persisted, and robot-assisted stereotactic abscess drainage was performed. A culture of pus grew methicillin-resistant *Staphylococcus epidermidis* (MRSE). Whole-genome sequencing confirmed the species and ST2 subtype and genetic features associated with β-lactam resistance. Initially, based on the consultation findings, meropenem and metronidazole were empirically administered as broad-spectrum antibiotics; after MRSE was confirmed, treatment was switched to vancomycin, and the patient’s clinical symptoms and laboratory parameters gradually improved. Postoperative imaging showed no recurrence of abscess, and the patient was in satisfactory general condition and no aftereffects whatsoever at six months’ follow-up.

**Conclusion:**

We present a case report of an isolated brain abscess due to *Staphylococcus epidermidis* that developed after EVT of a ruptured intracranial aneurysm, which is a novel and clinically rare case. It also provides new clinical evidence for understanding this type of rare complication and optimizing treatment strategies.

## 1 Introduction

SAH is an acute cerebrovascular disease caused by the rupture of IAs. Studies have indicated that the mortality rate can be as high as 45% (1, 2). EVT has become an important therapeutic strategy for IAs with the development of neurointerventional techniques. However, EVT also has risks. Common complications include thrombus formation, distal embolization, intraoperative or postoperative hemorrhage, vasospasm, vascular perforation, and vascular dissection (3). Complications reported with aneurysm embolization, mechanical thrombectomy, and arteriovenous malformation Onyx embolization includes brain abscesses, but these are exceedingly rare (3–5). A recent review from 2025 reported cases of brain abscess following endovascular treatment of intracranial aneurysms, but only 11 cases were included (6).

A brain abscess is a type of focal suppurative inflammation due to infection. The pathogenesis involves the entrance of pathogens into the central nervous system, the breakdown of the blood-brain barrier(BBB), and the imbalance of the local immune response. SAH is a potential source of immune-inflammatory mechanisms. The SAH causes not only mechanical brain injury but also can trigger a systemic inflammatory response, which puts the body in a state of immunosuppression and increases the risk of infection. Stents can also cause a vascular inflammatory response, resulting in endothelial injury, platelet activation, recruitment of inflammatory cells, and release of inflammatory factors. This condition may impair the integrity of the vascular endothelium, the BBB function, and neuroimmune homeostasis, thus providing a milieu in which bacteria can enter brain tissue (7). *Staphylococcus epidermidis* is a Gram-positive, coagulase-negative staphylococcus belonging to the genus Staphylococcus. It typically colonizes the human skin, nasal cavity, and other mucosal surfaces and has long been considered an important component of the normal human microbiota; however, under specific clinical conditions, it can become an opportunistic pathogen. In this article, we report a case of a *Staphylococcus epidermidis*-associated brain abscess following EVT of a ruptured intracranial aneurysm; a review of the relevant literature reveals that such cases are extremely rare. We also further analyze the potential immune and inflammatory mechanisms underlying this case, as well as treatment strategies for brain abscesses.

## 2 Case report

### 2.1 First hospitalization

A 49-year-old male patient, with no previous medical history, was admitted to the hospital emergency department with headaches, seizures, vomiting, urinary incontinence, and persistent drowsiness for almost one day. The patient had no history of hypertension or diabetes, nor did he have a history of smoking, alcohol consumption, travel, or prior medication use. His family history was unremarkable. On admission, the patient’s temperature was 37.1 °C, pulse rate was 85 beats per minute, and blood pressure was 155/91 mmHg. The patient was somnolent and presented with positive nuchal rigidity. Laboratory tests revealed that serum electrolytes, liver function, renal function, and coagulation parameters were generally within normal ranges. However, the white blood cell count (14.60 × 10^9^/L) and granulocyte count (12.30 × 10^9^/L) were markedly elevated, suggesting a possible infection. The patient tested negative for infectious diseases and had no history of HIV or syphilis. A head CT scan revealed subarachnoid hemorrhage (**Figure 1**), and a lung CT scan showed mild consolidation-like changes in both lungs. A head and neck CTA revealed a saccular aneurysm in the anterior communicating artery (**Figure 1**), measuring approximately 5.8 mm × 5.6 mm, with a neck width of 3.6 mm and an irregular shape accompanied by a subaneurysmal protrusion; this was considered the cause of the SAH.

**Figure 1:**
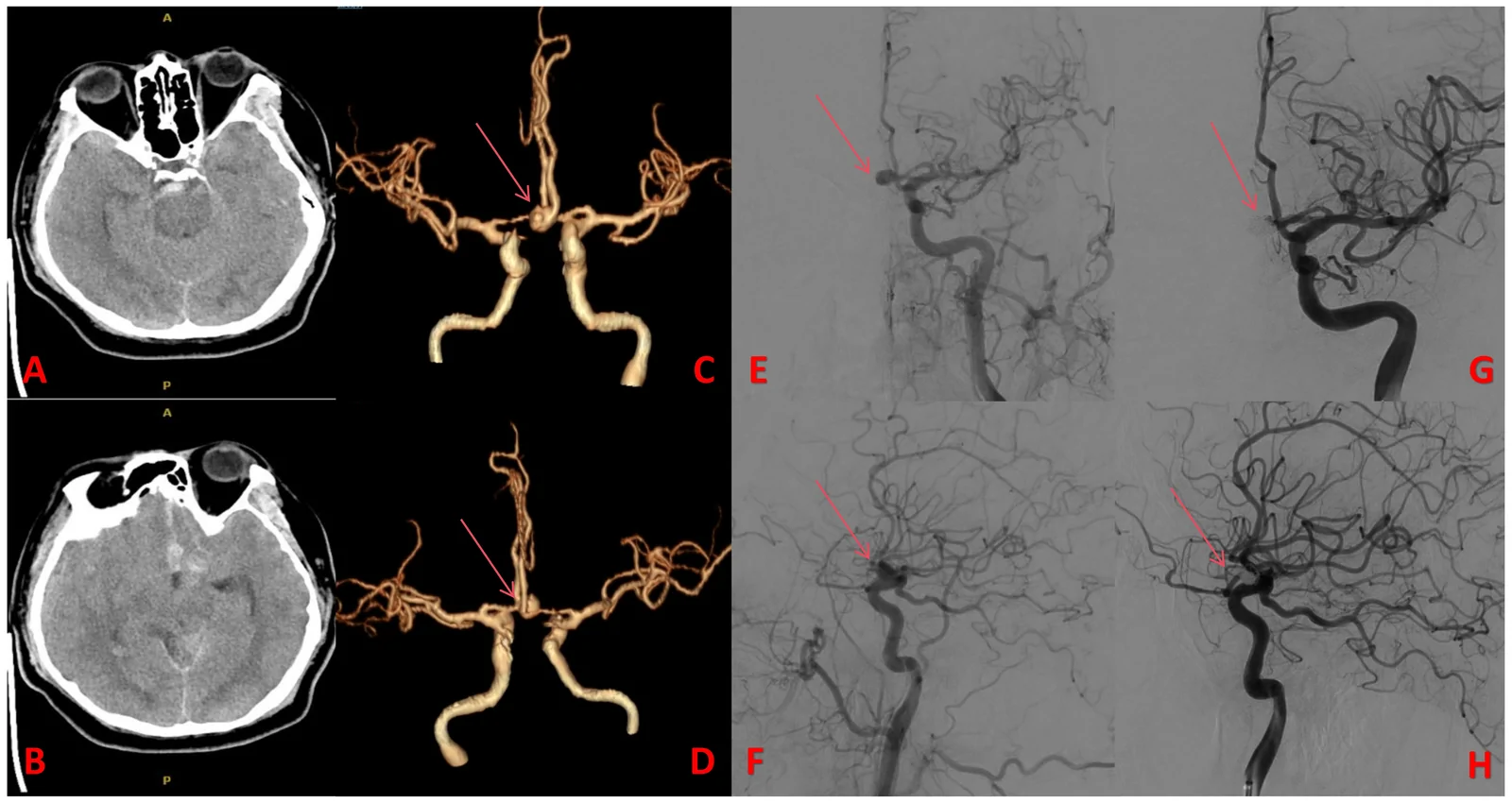
Initial CT **(A, B)**, CTA **(C, D)** and DSA **(E-H)** at Admission. **(A, B)** Multiple hyperechoic lesions are visible in the pre-pons pool, the suprasellar cistern, the falx cerebri, the sulci and fissures along the midline, and the bilateral frontal sulci and fissures. Strip-like, slightly hyperechoic lesions are visible in the left cerebellum and pons. **(C, D)** A sac-like aneurysmal change is visible on the right side of the anterior communicating artery. **(E, F)** The patient's preoperative 2D-DSA in the anteroposterior and lateral views confirmed an aneurysm of the anterior communicating artery. **(G, H)** Postoperative 2D-DSA in the anteroposterior and lateral views shows no opacification of the anterior communicating artery aneurysm.

On physical examination, the patient was somnolent, confused, and disoriented, with positive nuchal rigidity and a Hunt-Hess score of III. The patient underwent endovascular coiling of the aneurysm with stent support in the emergency department, the surgery lasts about 2 hours. An Atlas stent (3.5 mm × 15 mm) was used, and the first coil deployed measured 5 mm × 17 cm. The procedure proceeded smoothly, and immediate angiography demonstrated Raymond Class I embolization (**Figure 1**). Mannitol, nimodipine, steroids, and antiplatelet agents were routinely given as postoperative treatment. At the same time, ceftriaxone was routinely administered empirically for treatment of pulmonary inflammation. Sputum culture did not show any specific respiratory pathogens. The patient had an uneventful postoperative course except for a short episode of fever. The laboratory tests revealed a slight increase of inflammatory markers with leukocytes at 14.60 × 10^9^/L, C-reactive protein (CRP) at 3.30 mg/l, and a controlled procalcitonin at 0.037 ng/ml. A follow-up head and lung CT scan on Day 10 showed that the hemorrhage had largely resolved, and the pulmonary inflammation had improved. A follow-up examination on Day 13 revealed that the white blood cell count had returned to near-normal levels (10.58 × 10^9^/L), and CRP had decreased to 1.90 mg/L, and the remaining infection markers were also essentially normal (**Table 1**). The patient’s vital signs were stable, he was alert and oriented, and he showed no neurological deficits; he was discharged the following day. At discharge, the patient had no fever, nuchal rigidity was absent (–), and his modified Rankin Scale (mRS) score was 0. The patient received continuous anti-inflammatory treatment with ceftriaxone during hospitalization; given his favorable condition and laboratory results at discharge, anti-inflammatory therapy was not continued after discharge.

**Table 1:** Laboratory test results for the entire course of treatment.

Time point	Temperature(°)	Leukocytes(10^9/L)	Granulocytes(10^9/L)	Lymphocytes(10^9/L)	CD3+ T lymphocytes(10^9/L)	CD4+ T lymphocytes(10^9/L)	CD8+ T lymphocytes(10^9/L)	CD4/CD8	CD19+ B lymphocytes(10^9/L)	CD20+ B lymphocytes(10^9/L)	Natural killer cells(10^9/L)	IgG(g/L)	IgA(g/L)	IgM(g/L)	C3(g/L)	C4(g/L)	IL-1β(pg/mL)	IL-6(pg/mL)	TNF-α(pg/smL)	IL-10(pg/mL)	C-reactive protein(mg/L)	Procalcitonin(ng/ml)
Day3	37.1	14.60	12.30	1.46	——	——	——	——	——	——	——	——	——	——	——	——	——	——	——	——	——	——
Day6	38.3	20.75	18.57	0.75	——	——	——	——	——	——	——	——	——	——	——	——	——	——	——	——	4.10	0.052
Day10	37.4	14.92	10.89	2.91	——	——	——	——	——	——	——	——	——	——	——	——	9.6	22.4	15.1	8.5	3.30	0.037
Day13	36.6	10.58	7.47	2.41	——	——	——	——	——	——	——	——	——	——	——	——	4.5	8.9	9.2	4.6	1.90	0.025
Day34	38.9	16.38	13.55	0.88	——	——	——	——	——	——	——	——	——	——	——	——	——	——	——	——	3.60	0.068
Day37	38.5	17.65	15.81	0.63	0.44	0.13	0.30	0.43	0.06	0.05	0.09	16.3	3.7	2.8	0.68	0.14	17.2	90.5	57.8	31.2	4.90	0.075
Day41	37.4	11.42	9.88	0.98	——	——	——	——	——	——	——	——	——	——	——	——	——	——	——	——	2.60	0.049
Day44	36.8	9.65	7.27	1.12	0.78	0.38	0.39	0.97	0.14	0.12	0.20	13.4	4.1	1.9	1.10	0.26	7.3	32.4	23.0	12.2	1.60	0.041
Day46	36.4	8.82	4.41	3.63	——	——	——	——	——	——	——	——	——	——	——	——	——	——	——	——	2.50	0.032
Day50	36.5	7.97	3.73	2.84	2.05	1.19	0.83	1.43	0.28	0.25	0.34	10.3	2.9	1.2	1.35	0.34	3.8	13.2	10.5	5.8	1.80	0.028

### 2.2 Second hospitalization

On day 34, the patient developed a high fever with headache, nausea, and vomiting. Despite treatment with mannitol and ceftriaxone, the patient was not relieved and was re-admitted to our hospital’s emergency department. On admission, the patient’s temperature was 38.9 °C, pulse rate was 84 beats per minute, and blood pressure was 131/95 mmHg. The patient was alert and cooperative during the physical examination and showed no pathological signs. Laboratory tests showed normal CRP levels, but white blood cell count (16.28 × 10^9^/L), granulocyte count (13.55 × 10^9^/L), and procalcitonin (0.068 ng/mL) were significantly elevated, whereas lymphocyte count (0.88 × 10^9^/L) was decreased. Given the presence of systemic and neurological symptoms, an intracranial infection was suspected, and brain imaging was therefore performed. A head CT and head and neck CTA were performed in the emergency department (**Figure 2**). The CT revealed a nearly circular hypodense lesion in the left frontal lobe, accompanied by a ring-like area of slightly increased density and surrounding patchy hypodensity, strongly suggesting a brain abscess; the CTA showed no evidence of aneurysm recurrence.

**Figure 2:**
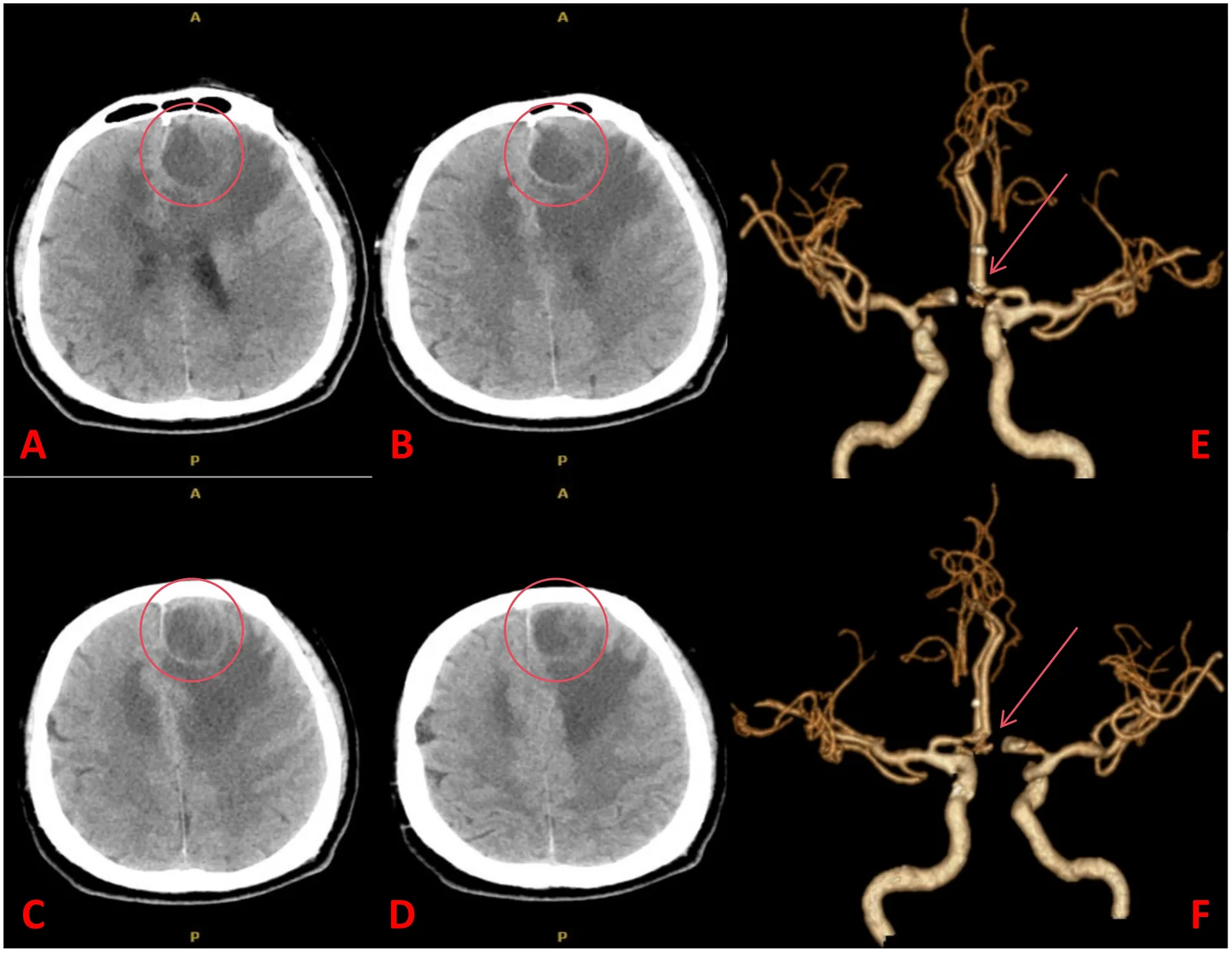
CT **(A–D)** and CTA **(E, F)** Scans During the Second Hospitalization. **(A, B)** Bilateral lobular low-density opacities in the frontal lobes, more pronounced on the left; blurring of the cerebral sulci; compression of the anterior horns of both lateral ventricles. **(E, F)** A dense shadow is seen within the anterior communicating artery; no abnormalities are noted in the anterior cerebral artery; post-interventional procedure on the anterior communicating artery.

After admission, the patient underwent a head MRI with plain and contrast-enhanced scans (**Figure 3**), which revealed a nearly circular lesion with mixed long T1 and long T2 signal intensity in the left frontal lobe, surrounded by a band-like area of long T2 signal edema, approximately 42 mm × 27 mm. On the contrast-enhanced scan, the lesion margins showed marked enhancement. At this time the patient reported more intense headaches and developed somnolence, disorientation, and weakness of the right-sided limbs. The immune status of the patient was assessed with a comprehensive immunological evaluation because he developed a brain abscess after aneurysmal subarachnoid hemorrhage and EVT in the absence of any known history of immunodeficiency. The patient’s CD3+T lymphocytes (0.44 × 10^9^/L), CD4+ T lymphocytes (0.13 × 10^9^/L), and CD8+ T lymphocytes (0.30 × 10^9^/L) were significantly below the normal range, while CD19+ B lymphocytes (0.06 × 10^9^/L), CD20+ B lymphocytes (0.05 × 10^9^/L), and natural killer cells (0.09 × 10^9^/L) were also reduced to varying degrees compared to the normal range. Complement C3 (0.68 g/L) and C4 (0.14 g/L) were also lower than normal, and levels of inflammatory markers—including IL-1β (17.2 pg/mL), IL-6 (90.5 pg/mL), TNF-α (57.8 pg/mL), IL-10 (31.2 pg/mL), and others were also significantly elevated (**Table 1**). We also performed blood and urine cultures, but the results were all negative. The patient’s lung CT scan also showed no significant abnormalities. Based on the available test results, no clear foci of extracranial infection were identified. In particular, there was no conclusive evidence of other systemic infection foci that could clearly explain the formation of the intracranial abscess. MRI findings revealed a ring-shaped enhancing lesion in the left frontal lobe, consistent with the characteristics of a brain abscess; however, the spatial resolution of the available imaging data was insufficient to determine whether there was a direct anatomical connection between the abscess cavity and the previously treated anterior communicating artery aneurysm or the implanted endovascular device.

**Figure 3:**
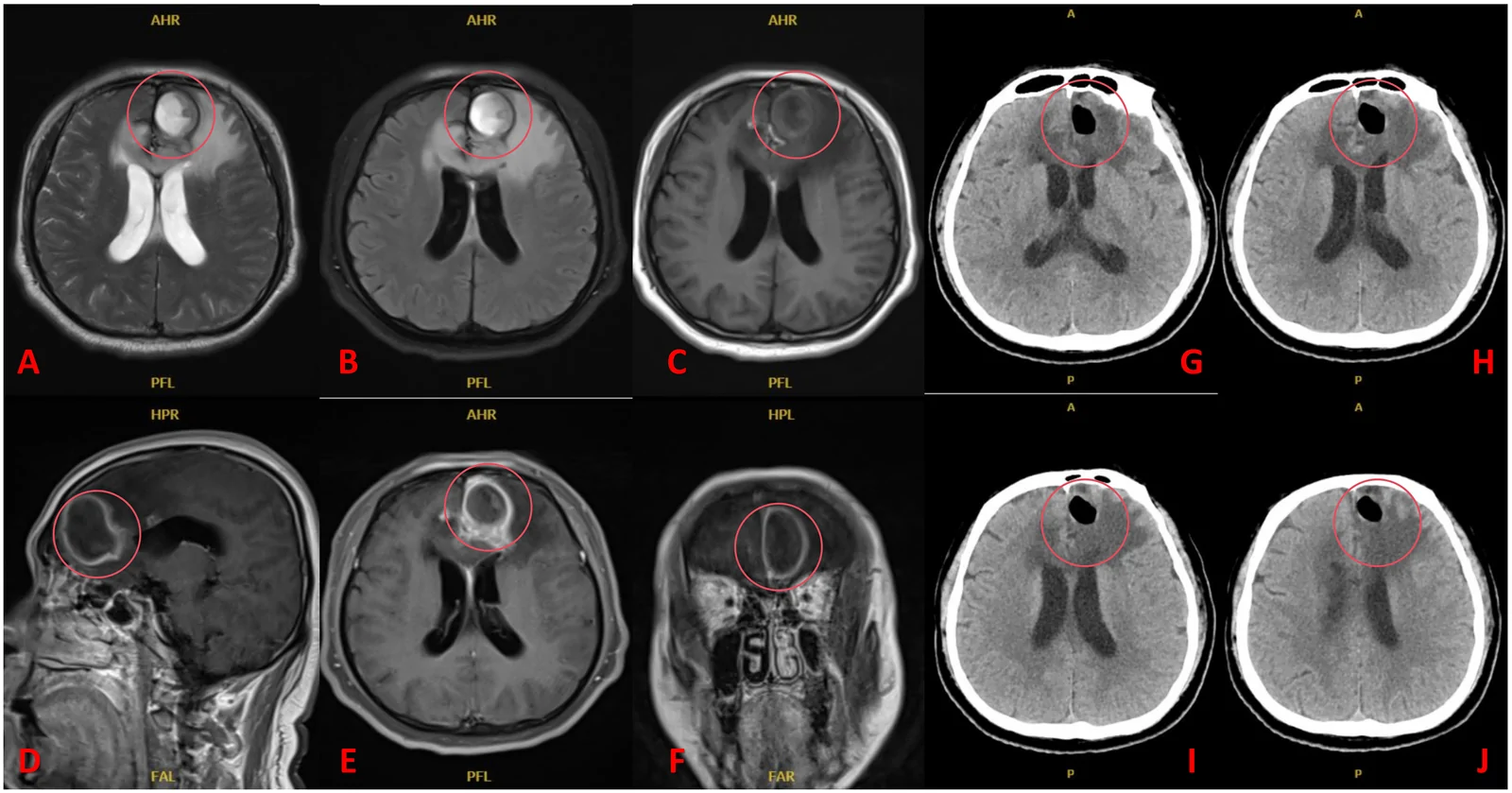
Preoperative contrast-enhanced MRI **(A–F)** and postoperative CT **(G–J)**. **(A–F)** Flat, elongated areas of T2-hyperintense edema are seen in the Anshang region and both frontal lobes, containing small, cord-like areas of T1-hypointense signal. A near-circular area of mixed T1- and T2-hyperintense signal is seen in the left frontal lobe, appearing as mixed high and low signal on FLAIR sequences. There is mild compression of the bilateral basal ganglia. **(G–J)** Patchy areas of low density are visible in both frontal lobes, with the left frontal lobe being more prominent; air-filled areas of high density are visible within these patches.

Given the patient’s current immune dysfunction and based on the recommendations of a multidisciplinary consultation, the patient was treated with meropenem and metronidazole for infection.

We reassess the patient’s condition 10 days later to document changes in the patient’s clinical course over time. Following the follow-up examination, all cell counts showed improvement, and inflammatory marker levels had also decreased significantly compared to the time of admission (**Table 1**). However, the patient’s symptoms—including headache, nausea, and vomiting—showed no significant improvement. Given that the patient’s current condition permitted it, a robot-assisted stereotactic abscess drainage procedure was performed under general anesthesia on day 45. During the procedure, approximately 15 mL of yellowish-brown, viscous fluid was drained until the abscess cavity became clear, successfully reducing the abscess burden. The drained fluid was sent for bacterial culture and identification. Results indicated that the causative pathogen was *Staphylococcus epidermidis*; no other pathogens were cultured. The organism was resistant to penicillin G, ampicillin, erythromycin, and clindamycin, with resistance mechanisms confirmed by a positive methicillin test and positive β- lactamase test. We adjusted the antibiotic regimen accordingly, adding vancomycin for infection control and discontinuing metronidazole. The patient’s condition continued to improve, and follow-up tests showed that white blood cell count (7.97 × 10^9^/L), granulocyte count (3.73 × 10^9^/L), lymphocyte count (2.84 × 10^9^/L), and procalcitonin (0.028 ng/mL) had all returned to normal ranges. We also re- evaluated other immune and inflammatory markers, all of which had returned to or were close to normal ranges. A follow-up head CT scan (**Figure 3**) showed no recurrence of the abscess, and the patient was discharged again on day 52. At discharge, the patient was afebrile and had an mRS score of 0.

Further characterization of the genomic features of the infecting strain, a *Staphylococcus epidermidis* strain isolated from brain abscess specimen, was done using whole genome sequencing (WGS). The strain was identified as *Staphylococcus epidermidis* by bacterial culture and MS identification. One pure colony was picked to expand the strain and extract bacterial genomic DNA. The species was identified as *Staphylococcus epidermidis* by average nucleotide identity analysis (≥95%). The Multilocus Sequence Typing (MLST) protocol was used for sequence typing. The Basic Local Alignment Search Tool was used to identify the strain as belonging to ST2. Resistance gene analysis confirmed the presence of genetic determinants of resistance to β-lactam antibiotics, as predicted by the methicillin-resistant phenotype determined by clinical susceptibility testing.

The patient underwent follow-up examinations one month and six months after discharge. No recurrence of the abscess or new symptoms were observed, and the patient was in generally good health with no residual neurological deficits. The timeline of the patient’s clinical course is shown in **Figure 4**. The laboratory test results during the patient’s hospitalization are shown in **Table 1**.

**Figure 4:**
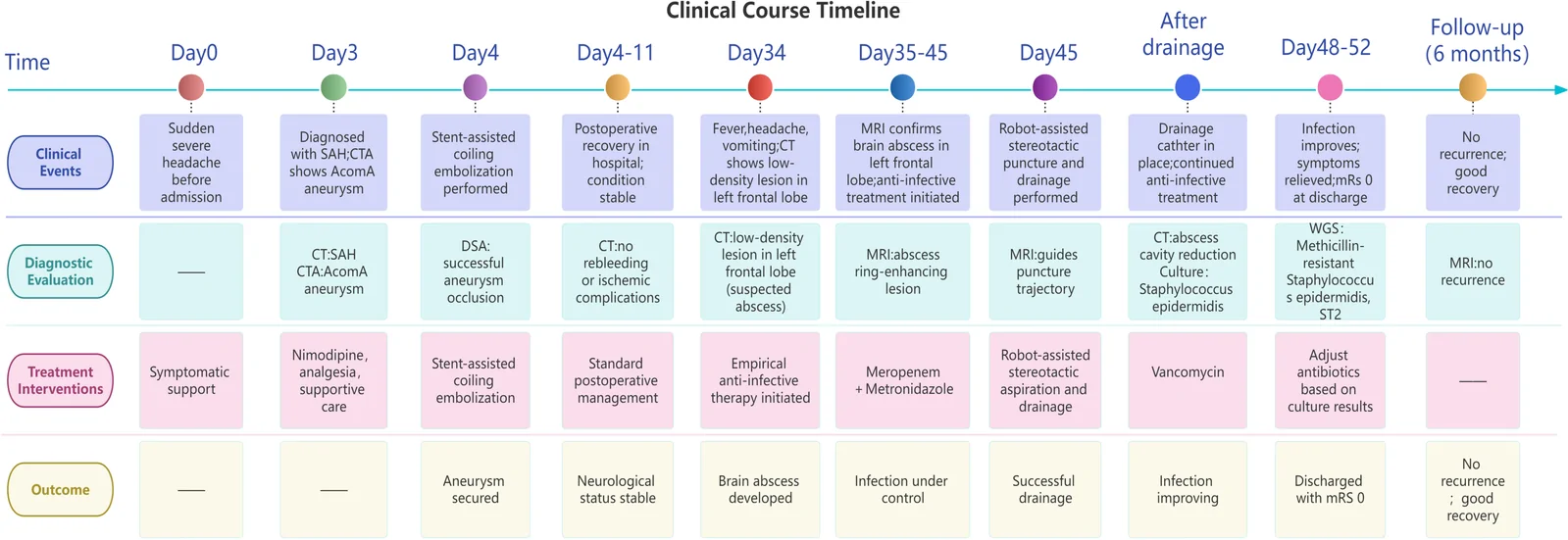
Clinical course timeline. SAH, subarachnoid hemorrhage; CTA, computer tomography angiography; AcomA, anterior communicating artery; DSA, digital subtraction angiography; MRS, magnetic resonance imaging; CT, computed tomography; mRS, modified Rankin Scale; WGS, whole-genome sequencing.

## 3 Discussion

### 3.1 Clinical challenges and the rarity of post-EVT brain abscesses

A brain abscess is a focal suppurative inflammation caused by the invasion of pathogens into the brain parenchyma. Its development typically involves multiple processes, including the spread of pathogens, disruption of the BBB, a local inflammatory response, and the formation of a capsule around the abscess cavity; this capsule is clearly visible on MRI images taken at the time of readmission (8). For patients undergoing neurovascular interventional procedures, brain abscess is not a common complication; however, a small number of previously reported cases suggest that brain abscesses may occur following aneurysm embolization, mechanical thrombectomy, and interventional treatment of cerebral vascular malformations. We, on the other hand, reported a case of a *Staphylococcus epidermidis*-associated brain abscess following EVT of a ruptured aneurysm (3–6).

Due to its low incidence and the fact that early postoperative symptoms can easily be confused with the underlying cerebrovascular disease, postoperative inflammatory response, or other infectious complications, clinical recognition presents certain challenges. Fever, headache, nausea and vomiting are symptoms which may suggest an intracranial infection, but these symptoms are not specific and may be overlapping with common complications or symptoms after aneurysmal SAH. Without appropriate neuroimaging, a brain abscess may not be immediately distinguishable from other causes of fever and neurological symptoms.

### 3.2 Potential sources of infection and hematogenous spread in brain abscesses

In this case, *Staphylococcus epidermidis* was directly isolated from a culture of pus from a brain abscess, and no other pathogens were cultured; therefore, there is strong evidence supporting its role as the causative agent of the brain abscess. However, the initial source of the infection remains undetermined. The patient showed no clear signs of a pulmonary or urinary tract infection; urine analysis and lung CT scans during hospitalization were largely normal, and relevant tests did not reveal any definite foci of distant infection. Therefore, the likelihood of direct spread is relatively low. However, femoral artery puncture, catheter procedures, intravascular stent implantation and perioperative contamination from the skin and medical device-associated colonization, may all be considered routes of infection (9, 10). We hypothesize that after bacteria briefly enter the bloodstream, they reach the brain tissue via the hematogenous route against a backdrop of altered host defense mechanisms and local barriers, ultimately leading to a focal infection; the integrity of the BBB may be a critical factor in this process (11). SAH, brain tissue damage, local inflammation, and microcirculatory disturbances may all affect the stability of the neurovascular unit, thereby increasing the brain tissue’s susceptibility to circulating pathogens and inflammatory mediators (12). Therefore, in this case, hematogenous spread and altered BBB function may jointly constitute the underlying basis for the formation of a brain abscess; however, this mechanism requires further research and validation in the future.

### 3.3 Dynamic changes in immune and inflammatory responses following SAH

Following SAH, the systemic immune and inflammatory responses can be robust, and they not only cause damage to brain tissue in a specific area. In the early stages of brain injury, blood in the subarachnoid space and its degradation products can activate microglia, astrocytes and cerebral vascular endothelial cells to release inflammatory mediators, including IL-1β, IL-6 and TNF-α, through signaling pathways involving pattern recognition receptors, NF-κB and inflammasomes (13, 14). However, the immune response following SAH is not simply a sustained increase in inflammation but may manifest as a dynamic process in which early inflammatory activation coexists with subsequent immune regulation or immunosuppression. Previous studies have shown that activation of the sympathetic nervous system and the hypothalamic–pituitary–adrenal axis following SAH can further influence the function of peripheral immune cells, leading to changes in T-cell responses, monocyte function, and the cytokine network (15). The laboratory results in this case are consistent with this dynamic change. At the time of the second hospitalization, most of the patient’s laboratory test results were outside the reference range; following antimicrobial therapy and abscess drainage, these values largely returned to normal. With regard to *Staphylococcus epidermidis*, this bidirectional immune response may significantly increase the risk of opportunistic infections.

### 3.4 Vascular implants induce an immune microenvironment

In addition to SAH itself, EVT and implant materials may further affect the local vascular microenvironment. Following stent implantation, the vascular endothelium must undergo a process involving adhesion, an inflammatory response, and gradual endothelialization. Contact between the stent surface and blood components can affect platelets and complement and may trigger a complex vascular immune response through endothelial cell activation and the recruitment of inflammatory cells. Indeed, during the patient’s second hospitalization, complement levels C3 and C4 did rise from low to high (16). In this case, it is currently impossible to determine whether the stent and coil were directly involved in the infection process. Particularly in the absence of culture results from the retrieved stent, histopathological evidence, or microbiological evidence from the device surface, the implanted material cannot be directly identified as the source of infection. However, from a biological perspective, the surface of a medical device may indeed serve as a potential niche for *Staphylococcus epidermidis* adhesion and biofilm formation (17). Therefore, EVT-related local inflammation is more appropriately considered a possible contributing factor rather than a definitive pathogenic mechanism.

### 3.5 Potential interactions between *Staphylococcus epidermidis* and the host immune response

In this case, *Staphylococcus epidermidis* was isolated from pus within a brain abscess cavity rather than from a peripheral blood culture alone. This clinical isolate was confirmed based on typical imaging findings of the abscess cavity, the clinical course of infection, the response to treatment, and WGS and was identified as ST2 through MLST analysis. Unlike the severe tissue destruction caused by highly virulent bacteria, *Staphylococcus epidermidis* infections are often characterized by a persistent, insidious course. Their impact on the nervous system depends not only on the biological characteristics of the bacteria themselves but is also closely related to an imbalance in the host’s neuroimmune homeostasis (18). A complex bidirectional regulatory relationship exists among brain tissue, the immune system, and microorganisms. Low-virulence staphylococci can maintain the infection in a state of low-level inflammatory equilibrium by modulating the release patterns of inflammatory cytokines, thereby avoiding rapid clearance by the host. This characteristic allows them to persist long-term and gain a pathogenic advantage when the host’s immune status changes (18, 19). However, the immune response exhibits a distinct dual effect. On the one hand, host immune activation helps limit bacterial spread and promotes clearance of the infection; on the other hand, excessive immune activation may cause additional damage, exacerbating brain tissue inflammation and neurological dysfunction (20, 21). Therefore, the formation of a brain abscess is not a simple process of bacterial proliferation but rather a dynamic process jointly determined by pathogen characteristics, the host’s immune status, and the inflammatory response (21).

### 3.6 Review of the literature

This study conducted a non-systematic, narrative literature search using PubMed and Web of Science to identify published reports on brain abscesses following endovascular treatment of intracranial aneurysms. The search employed a combination of the following terms: “brain abscess,” “intracranial aneurysm,” “cerebral aneurysm,” “endovascular treatment,” “embolization,” “coil embolization,” and “stent-assisted coil embolization.” As this was a narrative review, no formal systematic review protocol or study screening process based on PRISMA criteria was employed. Only six cases were identified in the literature regarding cerebral abscesses following EVT of ruptured aneurysms (**Table 2**); none of the patients in these reports mentioned a clear history of preoperative infection (4, 22–25).Among the seven cases, including the present one, the pathogen was not specified in 3 cases (4, 23, 24), while 3 cases were caused by Staphylococcus aureus and Salmonella (22, 25, 26). Only the present case involved *Staphylococcus epidermidis*, which may be the first reported case of this kind. In this case, the shortest duration for abscess formation was 20 days. Regarding location, this case was the only one involving an AcomA aneurysm. All cases received antimicrobial therapy; most had favorable outcomes, with only one death and two cases of varying degrees of disability (4, 23, 25).

**Table 2:** Literature review of brain abscess cases following endovascular treatment of ruptured intracranial aneurysms.

Year	Journal	Author	Age	Sex	Preoperative infection	Symptom 1	Size(mm)	Location	Surgery 1	Postoperative interval	Symptom 2	Surgery 2	Pathogen	Outcome
Present case	——	——	49	Male	Yes	Headache, nausea, and vomiting	5.8×5.6	AcomA	Stent-assisted coil embolization	20 days	Headache and high Fever	Robot-assisted stereotactic puncture and drainage surgery and antimicrobial therapy	Staphylococcus epidermidis	Recovery
2018	Surgical neurology international	Charles Fish, et al. (23)	72	Female	No	Traumatic subdural hematoma	5.5×3.4×3.7	R-ICA	Balloon-assisted coil embolization	1 month	Fever, left-sided limb paralysis, and disturbance of consciousness	Antimicrobial therapy	Not specified	Disability
2018	Interventional neuroradiology	Jung Ho Ko,et al. (22)	24	Male	No	Headache	1.5	L-MCA	Stent-assisted coil embolization	3 months	Headache, nausea, and vomiting	Stereotactic puncture and drainage surgery,antimicrobial therapy	Methicillin-resistant Staphylococcus aureus	Recovery
2017	Acta neurochirurgica	G. Cossu,et al. (24)	40	Female	No	Not specified	8×9×10	BA	Coil embolization	2 months	Headache, nausea, and vomiting	Abscess excision,external ventricular drainage,antimicrobial therapy	Not specified	Recovery
2014	Journal of Clinical Neuroscience	Guangzhong Chen,et al. (4)	54	Male	No	Not specified	7×6×5	L-MCA	Coil embolization	11 weeks	Headache	External drainage of the abscess	Not specified	Died
2003	Acta neurochirurgica	M D Jenkinson,et al. (25)	70	Female	No	Headache	Not specified	R-ICA	Coil embolization	5 weeks	Left-sided limb paralysis, and disturbance of consciousness	External ventricular drainage,antimicrobial therapy	Staphylococcus aureus	Disability
2002	Neurosurgery	Ramez W Kirollos,et al. (26)	55	Female	No	Not specified	Not specified	R-ICA	Coil embolization	3 years	Fever, cachexia, and vomiting	Antimicrobial therapy	Salmonella group D	Recovery

AcomA, anterior communicating artery; ICA, Internal carotid artery; BA, Basilar artery; MCA, Middle cerebral artery.

We also reviewed the literature on *Staphylococcus epidermidis*-associated brain abscesses, identifying a total of 8 cases, including the present one (**Table 3**) (6, 27–32). A summary of the reported cases revealed that patient ages ranged from 27 to 68 years, with males constituting the majority; most cases involved co-infection with other pathogens. Treatment typically included surgical intervention and antimicrobial therapy; in the present case, robot-assisted surgery was additionally performed. Of the 8 patients, half recovered (27, 29, 31), 2 died (6, 32), 1 was left with a disability, and the outcome for 1 was unknown (28, 30). Compared with some previous cases, this case involved a brain abscess with a single positive culture for *Staphylococcus epidermidis*, and the patient had no history of clear chronic underlying diseases. This further emphasizes the possible interactions between peri-procedural exposure, the host’s immunological status, and the characteristics of the pathogen itself.

**Table 3:** Literature review on Staphylococcus epidermidis-associated brain abscesses.

Year	Journal	Author	Age	Sex	Symptom	Co-pathogens	Treatment	Outcome
Present case	——	——	49	Male	Headache, nausea, and vomiting	——	Robot-assisted stereotactic puncture and drainage surgery and antimicrobial therapy	Recovery
2025	Surgical neurology international	José Airton Alves Ferreira, et al. (6)	51	Female	Headache and speech impairment	Staphylococcus hominis	Abscess drainage ,decompressive craniectomy and antimicrobial therapy	Died
2025	Surgical neurology international	Emilio González Martínez, et al. (27)	64	Female	Headache	Streptococcus mitis	Craniotomy and antimicrobial therapy	Recovery
2010	The American journal of the medical sciences	Patricia Cano, et al. (28)	54	Male	Left eye pain	Enterococcus faecalis	Craniotomy and antimicrobial therapy	Not specified
2010	The British journal of oral & maxillofacial surgery	P. Corre, et al. (29)	63	Female	Fever and disturbance of consciousness	Fusobacterium nucleatum	Stereotactic puncture and drainage surgery and antimicrobial therapy	Recovery
2007	Journal of chemotherapy	P. Malacarne, et al. (30)	27	Male	Headache and vomiting	——	Craniotomy and antimicrobial therapy	Disability
2005	Zhurnal voprosy neĭrokhirurgii imeni N. N. Burdenko	A N Konovalov, et al. (31)	Not specified	Male	Headache and fever	Nocardia brasilientis	Craniotomy and antimicrobial therapy	Recovery
1998	Acta neurochirurgica	M Pocar, et al. (32)	68	Male	Headache,fever and Left-sided hemiplegia	Staphylococcus species, Klebsiella pneumoniae and Enterobacter aerogenes	Craniotomy and antimicrobial therapy	Died

### 3.7 Discussion of treatment strategies

We also discussed the treatment strategies based on the relevant literature and our experience in treating this case. (1) Early detection and diagnosis: The incidence of brain abscess after EVT is generally very rare and easily overlooked, as the clinical presentation in this case was atypical. therefore, the development of an early identification system is crucial to improve prognosis. Unexplained fever, persistent headache, neurologic deficits, seizures, and elevated inflammatory markers in a patient after EVT should raise suspicion for a complication. CT is the typical initial neuroimaging modality for patients presenting with acute neurological symptoms and can demonstrate characteristic findings of a brain abscess, but early brain MRI should be obtained if CT is negative or if the diagnosis remains uncertain. (2) Antimicrobial therapy: from empirical coverage to targeted therapy. Post-EVT brain abscesses may require more extensive antimicrobial coverage. Cefepime or cefotaxime together with metronidazole can broaden the antimicrobial coverage against gram-negative bacteria (including Pseudomonas aeruginosa), and vancomycin can be added for methicillin-resistant gram-positive bacteria. Furthermore, when coverage against resistant Gram- positive bacteria is required, linezolid may be considered as an alternative to vancomycin. Current ESCMID guidelines conditionally recommend the use of carbapenems in combination with vancomycin or linezolid for post-neurosurgical brain abscesses, although the certainty of this evidence is low (33–35). However, in this case, since no staphylococcal infection or bacteremia was detected, and given that the source of infection and the causative pathogen remained unclear, the initial antimicrobial treatment regimen was designed to provide broad-spectrum coverage; therefore, vancomycin was not included in the initial empirical therapy. In retrospect, however, it would have been reasonable to consider incorporating vancomycin into the treatment regimen at an earlier stage. Therefore, the initial treatment regimen should be interpreted in light of the clinical information available at the time, rather than based on microbiological results obtained later.(3)Surgical Treatment. A traditional craniotomy can be performed for large abscesses with significant mass effects and edema. Nevertheless, when feasible, stereotactic drainage with robotic assistance may have some benefits, since it can precisely avoid functional areas, nerves, and blood vessels and enhance the accuracy of pathogen diagnosis and allow for rapid bacterial culture and antibiotic susceptibility testing. Extraventricular drainage may also be contemplated in patients with hydrocephalus, in whom the infection has spread to the ventricles, or with other conditions causing obstruction of cerebrospinal fluid or increased intracranial pressure. The patient initially presented with fever, headache, nausea, and vomiting without severe neurological deterioration, impairment of consciousness, or other signs of acute intracranial mass effect necessitating immediate decompression. Furthermore, the lesion was located in a relatively superficial region of the left frontal lobe; there was no ventricular rupture, no significant perilesional edema causing severe mass effect, and no radiological evidence suggesting an impending brain herniation. Therefore, surgery was not performed at the early stage.

### 3.8 Limitations

This case report has several limitations. First, as this is a single-case report, the mechanisms proposed in this paper—including altered BBB function, transient bacteremia, implant-related adhesion, and host immune and inflammatory changes—are primarily inferred from the clinical course and previous literature and require further experimental validation. Second, although *Staphylococcus epidermidis* was isolated from the pus of the brain abscess and further confirmed at the species level via WGS and MLST analysis, we were unable to obtain consecutive positive blood cultures or cultures from the stent or coil. Consequently, we could not determine the exact source of the infection nor prove that the intravascular implant itself was infected. Third, although this case showed decreased T-cell, B-cell, and NK-cell counts as well as elevated levels of various inflammatory cytokines, these changes occurred during the active phase of infection. Consequently, it is difficult to distinguish the independent contributions of factors such as SAH itself, infection, surgical stress, and drug therapy. Therefore, we interpret these findings as a transient immune- inflammatory imbalance rather than a primary immunodeficiency. Fourth, comprehensive transcriptomic analysis, virulence validation, or host–pathogen co-culture experiments have not yet been conducted. In the future, we will need to accumulate more case data and conduct integrated studies of host immunity, pathogen genomes, and functional phenotypes to further validate the mechanisms underlying the development of brain abscesses following EVT.

## 4 Conclusion

Compared with previous reports, this case presents an isolated *Staphylococcus epidermidis*- associated brain abscess that developed following EVT of a ruptured intracranial aneurysm. Successful treatment was achieved through robot-assisted stereotactic puncture and drainage combined with antimicrobial therapy, demonstrating a certain degree of clinical rarity and innovative value. Although a single case cannot prove a direct causal relationship between endovascular treatment, subarachnoid hemorrhage, and the immunological and inflammatory changes associated with the occurrence of a brain abscess, these factors may have played a role in precipitating the infection, and further studies are needed. This case provides new clinical evidence to understand this kind of rare complication and optimize treatment strategies.

## Data Availability

The raw data supporting the conclusions of this article will be made available by the authors, without undue reservation.
